# A curated transcriptome dataset collection to investigate the blood transcriptional response to viral respiratory tract infection and vaccination.

**DOI:** 10.12688/f1000research.18533.1

**Published:** 2019-03-13

**Authors:** Salim Bougarn, Sabri Boughorbel, Damien Chaussabel, Nico Marr

**Affiliations:** 1 Systems Biology and Immunology Department, Sidra Medicine, Doha, Qatar

**Keywords:** Transcriptomics, Bioinformatics, Software, Viral respiratory infection, Influenza viruses, Respiratory syncytial viruses (RSV), Rhinoviruses, Whole Blood, PBMC.

## Abstract

The human immune defense mechanisms and factors associated with good versus poor health outcomes following viral respiratory tract infections (VRTI), as well as correlates of protection following vaccination against respiratory viruses, remain incompletely understood. To shed further light into these mechanisms, a number of systems-scale studies have been conducted to measure transcriptional changes in blood leukocytes of either naturally or experimentally infected individuals, or in individual’s post-vaccination. Here we are making available a public repository, for research investigators for interpretation, a collection of transcriptome datasets obtained from human whole blood and peripheral blood mononuclear cells (PBMC) to investigate the transcriptional responses following viral respiratory tract infection or vaccination against respiratory viruses. In total, Thirty one31 datasets, associated to viral respiratory tract infections and their related vaccination studies, were identified and retrieved from the NCBI Gene Expression Omnibus (GEO) and loaded in a custom web application designed for interactive query and visualization of integrated large-scale data. Quality control checks, using relevant biological markers, were performed. Multiple sample groupings and rank lists were created to facilitate dataset query and interpretation. Via this interface, users can generate web links to customized graphical views, which may be subsequently inserted into manuscripts to report novel findings. The GXB tool enables browsing of a single gene across projects, providing new perspectives on the role of a given molecule across biological systems in the diagnostic and prognostic following VRTI but also in identifying new correlates of protection. This dataset collection is available at: http://vri1.gxbsidra.org/dm3/geneBrowser/list.

## Introduction

Viral respiratory tract infections (VRTI) are responsible for the majority hospitalizations among infants and the elderly. They are caused mainly by a heterogeneous group of viruses, including rhinoviruses, influenza viruses, parainfluenza viruses, respiratory syncytial virus (RSV), enteroviruses, coronaviruses, and certain strains of adenovirus ^1, 2^. Few antiviral therapies are currently approved and routinely used for VRTI. Most of these are specific inhibitors of influenza viruses ^3^. Moreover, for most respiratory viruses, there is no licensed vaccine available ^4, 5^, with the exception of flu vaccines for which protection generally lasts only one flu season. Consequently, clinical management of individuals with VRTI is mostly restricted to supportive care ^5^.

As clinical symptoms are often overlapping and are not specific for any of the viral species, it is difficult to establish a clinical diagnosis without laboratory testing ^1^. Furthermore, clinical manifestations of VRTI are highly variable, ranging from asymptomatic infections or illness with mild symptoms (a common cold) to clinically severe disease with life-threatening complications, such as respiratory failure and in some cases may have a fatal outcome ^6^. Infants, the elderly and patients with chronic lung or heart diseases in particular are at high risk ^7^.

Thus, there is an evident need to better understand the molecular mechanisms underlying the disease pathogenesis, progression as well as severity of, and immunity against, VRTI among humans ^8^. In this context, different large scale gene expression studies have been conducted using whole blood or peripheral blood mononuclear cells (PBMCs), to assess the human immune response to natural ^9– 11^ and experimental viral respiratory infections ^12, 13^; in particular, to influenza and RSV infections, and also to vaccination ^14– 17^.

Here, we make available, through an interactive web application, a curated collection of datasets that were obtained from pediatric and adult patients with natural VRTI, volunteers with experimental exposition to respiratory viruses and also vaccinated volunteers. Transcriptomics datasets were obtained from whole blood and PBMCs.

A total of 31 datasets were retrieved and selected from the NCBI Gene Expression Omnibus (GEO), a public repository of transcriptome profiles. The identified datasets are particularly relevant to our interest in understanding the pathobiology of VRTI and vaccination. As described in recent publications ^18, 19^, these datasets were loaded into a custom interactive web application, the Gene Expression Browser (GXB), which enables easy access to large datasets and interactive visualization of our dataset collection related to VRTI and vaccination against respiratory viruses. It also provides access to demographic and clinical information. Importantly, the user can customize data plots by adding multiple layers of parameters (e.g. age, gender, sample type, type of infection, type of vaccine, sample collection time), modify the sample ordering and genes, and generate links (mini URL) that can be shared via e-mail or used in publications. Therefore, we are providing here a resource enabling browsing of datasets relevant to blood transcriptional responses to VRTI and vaccination that offers a unique opportunity to identify host genes and their regulation that may be of diagnostic and/or prognostic value, or that may be tested as novel correlates of protection in subsequent studies. For example, a comparative approach of the transcriptional response signatures between experimentally infected and vaccinated individuals could be used to identify common mechanisms that define the poor health outcomes versus strong protection. The ability to pool, compare and analyze the immune responses to different infections and vaccines, in different individuals and at various age, offers a unique opportunity for a better understanding of the pathophysiology of VRTI.

## Methods

A total of 120 datasets, potentially relevant to human immune responses to VRTI and vaccination, were identified in GEO using the following search query:

Homo sapiens[Organism] AND ((“respiratory syncytial virus”[DESC] OR RSV[DESC] OR metapneumovirus[DESC] OR hMPV[DESC] OR influenza[DESC] OR parainfluenza[DESC] OR rhinovirus[DESC] OR rhinoviruses[DESC] OR adenovirus[DESC] OR adenoviruses[DESC] OR HAdV[DESC] OR coronaviruses[DESC] OR HCoV[DESC]) OR (vaccine OR vaccines OR vaccination) AND (blood[DESC] OR PBMC[DESC] OR PBMCs[DESC] OR lymphocyte[DESC] OR lymphocytes[DESC] OR “B cell”[DESC] OR “B cells”[DESC] OR “plasma cells”[DESC] OR “T cell”[DESC] OR “T cells”[DESC] OR Treg[DESC] OR Tregs[DESC] OR monocyte[DESC] OR monocytes[DESC] OR dendritic[DESC] OR DC[DESC] OR DCs[DESC] OR "natural killer"[DESC] OR NK[DESC] OR NKT[DESC] OR neutrophil[DESC] OR neutrophils[DESC]) AND (“Expression profiling by array”[gdsType] OR “Expression profiling by high throughput sequencing”[gdsType]).

**Figure 1.**
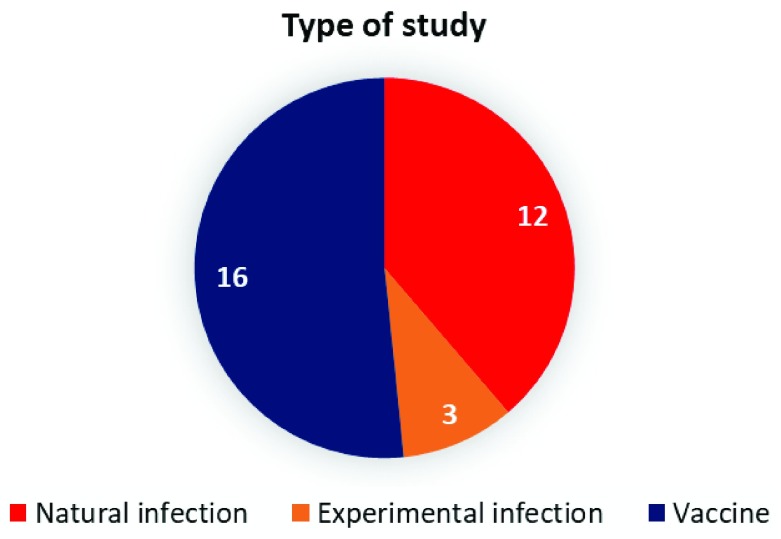
Break down of the dataset collection by category. The pie chart indicates the type of studies carried out for the 31 datasets.

Most of retrieved datasets were generated from human blood and human PBMC, using Illumina or Affymetrix commercial platforms or RNA-sequencing. All the entries that were returned with this query were manually curated. The process involved reading all the descriptions available of the datasets, the study design and the GEO-linked article in pubmed. Finally, only studies using human whole blood and human PBMCs, associated with natural or experimental VRTI, or vaccination against VRTI, were retained for our dataset collection. For the retained datasets, if the platform used to generate the transcriptome profiles was not supported by GXB or if from an *in vitro* study, they were exlcluded from our dataset collection. Based on these criteria, 31 datasets were retained. These include datasets that were generated from whole blood or PBMCs of individuals who were either naturally (12) or experimentally infected (3) (with influenza viruses, RSV, Rhinovirus, Rotavirus) as well as from healthy, uninfected (age-matched) volunteers. The remaining 16 datasets were generated from whole blood or PBMCs of individuals who had received flu vaccines ( Figure 1). The datasets that comprise our collection are listed in Table 1.

**Table 1.** List of datasets constituting the collection.

Title	Platforms used	Response to	Virus/Vaccine	Cell type/ Tissues	Number of samples	Selected marker for QC *	Citation #	GEO ID
Blood transcriptome of human bacterial and influenza A pneumonia.	Illumina HumanHT-12 v3	Natural infection	Influenza H1N1	Whole blood	190	Xist	9	GSE40012
Expression profiling of critically ill influenza and bacterial pneumonia patients, also influenza vaccination recipients.	Illumina HumanHT-12 v3	Natural infection and vaccination	Influenza H1N1	Whole blood	81	ND *	14	GSE20346
FACS-sorted cells from Young Adults Vaccinated with Influenza TIV or LAIV Vaccines during 2008/09 Flu Season	Affymetrix HG- U133A	Vaccination	Influenza TIV vaccine	PBMC	84	ND *	16	GSE29618
Gene expression analysis in children with complex seizures by influenza A (H1N1)pdm09 or rotavirus gastroenteritis.	Affymetrix HG U133_Plus2	Natural infection	Influenza (H1N1) Rotavirus	Whole blood	32	Xist	10	GSE50628
Gene expression analysis of Influenza vaccine response in Young and Old - Year 1.	Illumina HumanHT-12 v4	Vaccination	Influenza TIV vaccine	PBMC	72	ND *	23	GSE59635
Gene expression analysis of Influenza vaccine response in Young and Old - Year 2	Illumina HumanHT-12 v4	Vaccination	Influenza TIV vaccine	PBMC	156	ND *	23	GSE59654
Gene expression signatures of symptomatic respiratory viral infection in adults.	Affymetrix HG- U133A	Experimental infection	Influenza (H3N2) RSV Rhinovirus	Whole blood	113	ND *	13	GSE17156
Genome-wide analysis of whole blood transcriptional response to Respiratory Syncytial Virus (RSV), Influenza and Rhinovirus lower respiratory tract infection (LRTI) in children.	Illumina HumanHT-12 v4	Natural infection	Influenza, RSV, rhinovirus	Whole blood	36	Xist	8	GSE38900 (GPL10558)
Genome-wide analysis of whole blood transcriptional response to Respiratory Syncytial Virus, Influenza and Rhinovirus lower respiratory tract infection (LRTI) in children.	Illumina HumanWG-6 v3	Natural infection	Influenza, RSV, rhinovirus	Whole blood	205	Xist	8	GSE38900 (GPL6884)
Genome-wide profiling of whole blood from healthy adult volunteers before and after receiving non-live vaccines including seasonal influenza or pneumococcal vaccine or placebo (saline) injections I.	Illumina HumanHT-12 v3	Vaccination	Influenza TIV vaccine	Whole blood	72	Xist	24	GSE30059
Genome-wide profiling of whole blood from healthy adult volunteers before and after receiving non-live vaccines including seasonal influenza or pneumococcal vaccine or placebo (saline) injections II. Finger prick-Training Set	Illumina HumanHT-12 v3	Vaccination	Influenza TIV vaccine	Whole blood	185	Xist	24	GSE48762
Genome-wide profiling of whole blood from healthy adult volunteers before and after receiving non-live vaccines including seasonal influenza or pneumococcal vaccine or placebo (saline) injections II. Venipuncture-Training Set.	Illumina HumanHT-12 v3	Vaccination	Influenza QIV vaccine	Whole blood	214	Xist	24	GSE48762
Genome-wide profiling of whole blood from healthy adult volunteers before and after receiving non-live vaccines including seasonal influenza or pneumococcal vaccine or placebo (saline) injections II. Venipuncture-Validation Set.	Illumina HumanHT-12 v3	Vaccination	Influenza QIV vaccine	Whole blood	30	Xist	24	GSE48762
Global Analyses of Human Immune Variation Reveal Baseline Predictors of Postvaccination Responses.	Affymetrix HuGene 1.0 ST v1	Vaccination	Influenza QIV vaccine	PBMC	292	ND *	17	GSE47353
Host gene expression signatures of influenza A H1N1 and H3N2 virus infection in adults.	Affymetrix HG-U133A	Experimental infection	Influenza H1N1, H3N2	Whole blood	649	ND *	12	GSE52428
Host transcriptional response to influenza and other acute respiratory viral infections – a prospective cohort study.	Illumina HumanHT-12 v4	Natural infection	Influenza H1N1	Whole blood	880	Xist	11	GSE68310
Hosts responses in critical disease caused by pandemic H1N1.	Illumina HumanWG-6 v2	Natural infection	Influenza H1N1	Whole blood	40	ND *	25	GSE21802
Influenzavirus serotype association to global whole blood transcriptional changes.	Illumina HumanHT-12 v4	Natural infection	Influenza H1N1, H3N2	Whole blood	465	ND *	NA	GSE29385
Olfactomedin 4 serves as a marker for disease severity in pediatric Respiratory Syncytial Virus (RSV) infection.	Affymetrix HG-U133_Plus_2	Natural infection	RSV	PBMC	43	ND *	26	GSE69606
Patient-based transcriptome-wide analysis identify interferon and ubiquination pathways as potential predictors of Influenza A disease severity.	Illumina HumanHT-12 v4	Natural infection	Influenza H1N1, H3N2	Whole blood	402	ND *	27	GSE61821
Peripheral blood cells expression data form 7 patients with severe pdm(H1N1) influensa and 7 gender and age matched healthy controls.	Affymetrix HuGene 1.0 ST v1	Natural infection	Influenza H1N1	Whole blood	21	ND *	28	GSE27131
Systems biology of vaccination for seasonal influenza in humans.	Affymetrix HT_HG-U133_Plus_PM v1	Vaccination	Influenza TIV, LAIV vaccines	PBMC	163	ND *	16	GSE29619
Systems biology of vaccination for seasonal influenza in humans.	Affymetrix HG-U133A	Vaccination	Influenza TIV, LAIV vaccine	PBMC	84	ND *	16	GSE29619 (GPL3921)
T cell responses to H1N1v and a longitudinal study of seasonal influenza vaccination - 2009.	Illumina HumanHT-12 v4	Vaccination	Influenza QIV vaccine	Whole blood	72	ND *	NA	GSE58943
T cell responses to H1N1v and a longitudinal study of seasonal influenza vaccination - 2010.	Illumina HumanHT-12 v4	Vaccination	Influenza TIV vaccine	Whole blood	75	Xist	NA	GSE58898
T cell responses to H1N1v and a longitudinal study of seasonal influenza vaccination - 2012.	Affymetrix PrimeView v1	Vaccination	Influenza TIV vaccine	Whole blood	71	Xist	NA	GSE64514
Time Course of Adults Vaccinated with Influenza TIV Vaccine 2009-2012.	Affymetrix HT_HG-U133_Plus_PM v1	Vaccination	Influenza TIV vaccine	Whole blood	621	ND *	15	GSE74817
Time series of global gene expression after trivalent influenza vaccination in humans (female cohort).	Illumina HumanHT-12 v4	Vaccination	Influenza TIV vaccine	Whole blood	417	Xist	29	GSE48023
Time series of global gene expression after trivalent influenza vaccination in humans (male cohort).	Illumina HumanHT-12 v3	Vaccination	Influenza TIV vaccine	Whole blood	431	Xist	29	GSE48018
Transcriptional profile of PBMCs in patients with acute RSV or Influenza infection.	Illumina HumanHT-12 v4	Natural infection	Influenza (H1N1, H3N2), RSV	PBMC	101	Xist	30	GSE34205
Transcriptomic profiling facilitates classification of response to influenza challenge	Illumina HumanHT-12 v4	Experimental infection	Influenza H3N2	Whole blood	88	ND *	31	GSE61754
Transcriptomic profiling in childhood H1N1/09 influenza reveals reduced expression of protein synthesis genes	Illumina HumanHT-12 v3	Natural infection	Influenza (H1N1), RSV	Whole blood	92	Xist	32	GSE42026
Trivalent Inactivated Influenza Vaccine (TIV) and Live Attenuated Influenza Vaccine (LAIV) Induce Different B cell and Transcriptional Responses in Children	Illumina HumanHT-12 v4	Vaccination	Influenza TIV, LAIV vaccines	Whole blood	140	ND *	30	GSE52005
Whole Blood Transcriptional Response to Pediatric Influenza Infection	Illumina HumanWG-6 v3	Natural infection	Influenza	Whole blood	31	Xist	8	GSE29366

*Gender information and/or *Xist* probe was not available. QC – quality control, ND – no data

Once the final selection had been made, each dataset was downloaded from GEO by using the SOFT file format. Then, the datasets were uploaded on the Gene Expression Browser (GXB), an interactive web application hosted on the Amazon Web Services cloud ^20^. Information about samples and study design were also uploaded. The available samples were put into groups based on relevant study variables and genes were ranked according to the different groups comparisons. A detailed description of the GXB software tool is available from recent publications ^19– 21^. This software interface allows user to easily navigate and filter the dataset collection. A web tutorial can be easily accessed online. Annotation and functionality of the web software interface were described previously by our group ^18, 19, 21^, and is reproduced here so that readers can use this article as a standalone resource. Briefly, datasets of interest can be quickly identified either by filtering on criteria from pre-defined sections on the left or by entering a query term in the search box at the top of the dataset navigation page. Clicking on one of the studies listed in the dataset navigation page opens a viewer designed to provide interactive browsing and graphic representations of large-scale data in an interpretable format. This interface is designed to present ranked gene lists and display expression results graphically in a context-rich environment. Selecting a gene from the rank ordered list on the left of the data-viewing interface will display its expression values graphically in the screen’s central panel. Directly above the graphical display drop down menus give users the ability: a) To change how the gene list is ranked - this allows the user to change the method used to rank the genes, or to only include genes that are selected for specific biological interest; b) To change sample grouping (Group Set button) - in some datasets, a user can switch between groups based on cell type to groups based on disease type, for example; c) To sort individual samples within a group based on associated categorical or continuous variables (e.g. gender or age); d) To toggle between the bar chart view and a box plot view, with expression values represented as a single point for each sample. Samples are split into the same groups whether displayed as a bar chart or box plot; e) To provide a color legend for the sample groups; f) To select categorical information that is to be overlaid at the bottom of the graph - for example, the user can display gender or smoking status in this manner; g) To provide a color legend for the categorical information overlaid at the bottom of the graph; h) To download the graph as a portable network graphics (png) image. Measurements have no intrinsic utility in absence of contextual information. It is this contextual information that makes the results of a study or experiment interpretable. It is therefore important to capture, integrate and display information that will give users the ability to interpret data and gain new insights from it. We have organized this information under different tabs directly above the graphical display. The tabs can be hidden to make more room for displaying the data plots, or revealed by clicking on the blue “show info panel” button on the top right corner of the display. Information about the gene selected from the list on the left side of the display is available under the “Gene” tab. Information about the study is available under the “Study” tab. Rolling the mouse cursor over a bar chart feature while displaying the “Sample” tab lists any clinical, demographic, or laboratory information available for the selected sample. Finally, the “Downloads” tab allows advanced users to retrieve the original dataset for analysis outside this tool. It also provides all available sample annotation data for use alongside the expression data in third party analysis software. Other functionalities are provided under the “Tools” drop-down menu located in the top right corner of the user interface. Some of the notable functionalities available through this menu include: a) Annotations, which provides access to all the ancillary information about the study, samples and dataset organized across different tabs; b) Cross-project view; which provides the ability for a given gene to browse through all available studies; c) Copy link, which generates a mini-URL encapsulating information about the display settings in use and that can be saved and shared with others (clicking on the envelope icon on the toolbar inserts the URL in an email message via the local email client); d) Chart options; which gives user the option to customize chart labels.

## Quality Control

**Figure 2.**
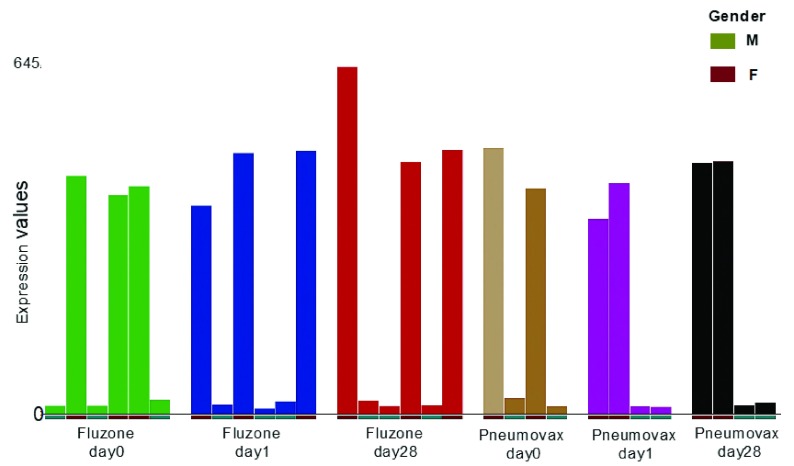
Shown are *XIST* gene expression levels and gender information from the venipuncture validation set of GSE48762. Gene expression data were from whole blood of healthy adult volunteers before and after receiving either placebo (saline) injections, seasonal influenza (Fluzone) or pneumococcal (Pneumovax) vaccination.

Quality control checks can be performed on the datasets loaded on GXB, for example by examining concordance of the gender-specific expression of the *XIST* gene in those datasets for which gender information was available as metadata. The *XIST* gene is essential for imprinted and random X-chromosome inactivation ^22^ and therefore, expression is expected to be high in female and low in male samples. Respective hyperlinks are found in Table 1 allow you to visualize the XIST experession based on the gender information provided with the GEO submission. Figure 2 shows *XIST* gene expression in a representative dataset, along with gender information available that was recorded and made available in GEO.

## Data availability

All datasets included in our curated collection are also available publically via the NCBI GEO website : https://www.ncbi.nlm.nih.gov/gds/; and are referenced throughout the manuscript by their GEO accession numbers (e.g. GSE17763). Signal files and sample description files can also be downloaded from the GXB tool under the “downloads” tab.
