# Supplementary material for: A curated transcriptome dataset collection to investigate the blood transcriptional response to viral respiratory tract infection and vaccination
Source: F1000Res. 2019 Mar 13;8:284. [Version 1] doi: 10.12688/f1000research.18533.1 (PMC6567289; doi:10.12688/f1000research.18533.1)
Supplement: Peer review history

# Peer review: Reviewer response for version 1

**DOI:** 10.5256/f1000research.20283.r48849

**Published:** 2019-06-13

**Authors:** Benjamin M Tang

**Affiliations:** 1 Department of Intensive Care Medicine, Nepean Hospital, Kingswood, NSW, Australia

This paper represents an extension of the authors' extensive work in this area - which aims to provide an accessible web-based platform for researchers to perform multi-cohort analysis of pooled transcriptomics data.

 The Introduction outlines the scientific rationale to support the development of such a platform, which I mostly agree. There are a number of reasons why such a platform can assist researchers, including (1) help researchers to leverage on publicly available dataset to address each researcher's own research question, (2) obtaining a large sample size for analysis, which is otherwise not feasible by a single research group, (3) provide access to a diverse range of patient population and varying degree of disease severity, thereby generating more biological insights, which are not achievable by a single-centre study. For these reasons, the platform provided by these author represents an important contribution to the research community.

 To further place this into perspective, one can estimate the likely cost (in time and money) to obtain the same analysis if such a platform is not available. To collect 31 transcriptomics datasets of viral respiratory infection (as outlined by this paper), a researcher need to perform an extensive literature review and database search. After all the datasets are collected, the researcher then need to bring in a bioinformatic team to perform a pooled analysis, which required advanced expertise due to the huge variability in technical platform, assay methods and normalization approach among different datasets. This bioinofrmatic work is extensive - if such work is outsourced to an external bioinformatician/company, the cost is in excessive of US$15,000 (our group has commissioned similar work in the past - hence our knowledge into the estimated cost). Here, the authors provides access to the analysis platform to the wider research community for free...an important step in democratising scientific data for the benefit of all researchers.

I have read this submission. I believe that I have an appropriate level of expertise to confirm that it is of an acceptable scientific standard.

# Peer review: Reviewer response for version 1

**DOI:** 10.5256/f1000research.20283.r45689

**Published:** 2019-03-26

**Authors:** Carlos Alberto Moreira-Filho

**Affiliations:** 1 Department of Pediatrics, Faculty of Medicine, University of São Paulo (USP), São Paulo, Brazil

The paper by Bougarn et al. describes the use of an interactive web application, GXB (Speake et al.(2015) ^1^), for investigating the blood transcriptional response to viral respiratory tract infection and vaccination. A curated dataset collection was established for this purpose encompassing 31 datasets retrieved and selected from the NCBI Gene Expression Omnibus (GEO) public repository.

   The rationale for creating the dataset collection and the methodology used in the paper are clearly described. The functionalities of the GXB tool are well explained to the reader, as well as the quality control procedures adopted by the authors. However, the GEO datasets do not always provide detailed information on virus genotypes. Distinct RSV genotypes induce very different PBMC transcriptional responses (see, for instance, Rodriguez-Fernandez et al. 2017 ^2^; and Vieira et al. 2019 ^3^). The authors could insert a comment on this issue in their paper.

I have read this submission. I believe that I have an appropriate level of expertise to confirm that it is of an acceptable scientific standard.

## References

[1] SpeakeCPresnellSDomicoKZeitnerBBjorkAAndersonDMasonMJWhalenEVargasOPopovDRinchaiDJourde-ChicheNChicheLQuinnCChaussabelD: An interactive web application for the dissemination of human systems immunology data. *J Transl Med*.2015;13: 10.1186/s12967-015-0541-x 196 10.1186/s12967-015-0541-x 26088622PMC4474328

[2] Rodriguez-FernandezRTapiaLYangCTorresJChavez-BuenoSGarciaCJaramilloLMoore-ClingenpeelMJafriHPeeplesMPiedraPRamiloOMejiasA: Respiratory Syncytial Virus Genotypes, Host Immune Profiles, and Disease Severity in Young Children Hospitalized With Bronchiolitis. *The Journal of Infectious Diseases*.2018;217(1) : 10.1093/infdis/jix543 24-34 10.1093/infdis/jix543 PMC585340729045741

[3] VieiraSEBandoSYde PaulisMOliveiraDBLThomazelliLMDurigonELMartinezMBMoreira-FilhoCA: Distinct transcriptional modules in the peripheral blood mononuclear cells response to human respiratory syncytial virus or to human rhinovirus in hospitalized infants with bronchiolitis. *PLoS One*.2019;14(3) : 10.1371/journal.pone.0213501 e0213501 10.1371/journal.pone.0213501 30845274PMC6405118
